# Coccidiosis in Egg-Laying Hens and Potential Nutritional Strategies to Modulate Performance, Gut Health, and Immune Response

**DOI:** 10.3390/ani14071015

**Published:** 2024-03-27

**Authors:** Milan Kumar Sharma, Woo Kyun Kim

**Affiliations:** Department of Poultry Science, University of Georgia, Athens, GA 30602, USA; milan.sharma@uga.edu

**Keywords:** coccidiosis, laying hens, gut health, oxidative stress, nutritional strategies, immune response, egg production

## Abstract

**Simple Summary:**

Coccidiosis is one of the most prevalent diseases in poultry production, inflicting substantial economic losses exceeding USD 15 billion to the poultry industry. Coccidiosis is most common in young broilers but is not limited to them, and pullets and egg-laying hens are equally susceptible. Unfortunately, unlike broilers, low emphasis has been given to laying hens. This review aims to summarize the effect of coccidiosis in laying hens while exploring potential nutritional interventions to fight coccidiosis.

**Abstract:**

Avian coccidiosis, despite advancements in management, nutrition, genetics, and immunology, still remains the most impactful disease, imposing substantial economic losses to the poultry industry. Coccidiosis may strike any avian species, and it may be mild to severe, depending on the pathogenicity of *Eimeria* spp. and the number of oocysts ingested by the bird. Unlike broilers, low emphasis has been given to laying hens. Coccidiosis in laying hens damages the gastrointestinal tract and causes physiological changes, including oxidative stress, immunosuppression, and inflammatory changes, leading to reduced feed intake and a drastic drop in egg production. Several countries around the world have large numbers of hens raised in cage-free/free-range facilities, and coccidiosis has already become one of the many problems that producers have to face in the future. However, limited research has been conducted on egg-laying hens, and our understanding of the physiological changes following coccidiosis in hens relies heavily on studies conducted on broilers. The aim of this review is to summarize the effect of coccidiosis in laying hens to an extent and correlate it with the physiological changes that occur in broilers following coccidiosis. Additionally, this review tries to explore the nutritional strategies successfully used in broilers to mitigate the negative effects of coccidiosis in improving the gut health and performance of broilers and if they can be used in laying hens.

## 1. Introduction

Avian coccidiosis, caused by various species of the protozoan *Eimeria*, poses a significant threat to the poultry industry. Despite advancements in management, nutrition, genetics, chemotherapy, and immunology, it remains the most prevalent disease in the poultry industry, imposing substantial economic losses of more than USD 15 billion [1]. The economic loss due to coccidiosis is not only due to clinical outbreaks resulting in low production and high mortality but also subclinical coccidiosis without visible clinical signs; even subclinical coccidiosis contributes to economic losses through reduced nutrient absorption and utilization, thereby compromising optimal performance and the feed conversion ratio (FCR). Reduced nutrient absorption during subclinical coccidiosis may result from intestinal enterocyte and morphometric damages, compromised gastrointestinal integrity, decreased ileal digestibility, and increased susceptibility to secondary diseases like necrotic enteritis [2,3,4,5,6,7].

Coccidiosis is common in young broilers or laying hen pullets ranging from 3 to 18 weeks of age but not limited to this age group. Newly hatched chicks have high maternal antibodies but have little protection against coccidiosis [8]. Chickens usually become infected when maternal antibodies deplete and the oocyst counts in litter peak at 3–6 weeks of age; however, immunity develops shortly after mild infections [2,5,8]. Older flocks without acquired immunity against *Eimeria* spp. might get the disease [2,8]. In addition, there is no cross-immunity among *Eimeria* species; thus, more than one outbreak is possible in the same flock with different species involved in each episode [2,8]. Generally, anticoccidial drugs are used in laying hens’ pullets and are withdrawn before they start laying eggs. Therefore, the acquired immunity against *Eimeria* during the pullet phase might not be enough to prevent infection throughout the production period [8,9,10]. Coccidiosis may strike any avian species, and it may be mild to severe, depending on the pathogenicity of the *Eimeria* spp. and the number of oocysts ingested by the bird [2,5]. 

Unlike broilers, limited attention has been given to laying hens regarding coccidiosis. One of the leading causes of laying hen mortality was reported as coccidiosis in cage-free systems [11,12,13]. A study conducted in Sweden observed 19% coccidiosis in laying hens raised on a litter-based cage-free system [14]. Laying hens recovered from coccidiosis but did not have optimal egg production and had a high FCR. Fossum et al. (2009) [12] observed higher incidences of coccidiosis in laying hens raised in cage-free systems (aviaries and free-range) compared with conventional cages in Sweden from 2001 to 2004, after the ban on conventional cages. Although there are several variations among cage-free systems (aviaries, free-range, or barn), the litter-based floor is typical in all housing systems that allow fecal–oral transmission of the oocyst, leading to disease outbreaks, unlike conventional cages. Therefore, preventing coccidiosis in these systems might become one of the many challenges for egg producers while transitioning from traditional cages to cage-free systems.

As the egg industry worldwide is steadily observing an increase in cage-free facilities, the broad topic of “gut health” associated with “coccidiosis” will have one of the most significant economic impacts on laying hen health and performance. Many countries in Europe have already transitioned to cage-free systems, whereas the United States is slowly transitioning to cage-free systems, and by 2023, more than 35% of laying hens in the U.S. were raised in cage-free environments, and this is still increasing (UEP, 2023). The evolution of these new production systems (aviaries; free-range) with large numbers of hens on the floor/range will influence the occurrence of coccidiosis. Laying hens raised in a litter-based aviary or free-range systems may provide a perfect environment for transmitting coccidiosis, and they have a higher chance of getting the disease than those raised in traditional cage systems. However, laying hens raised in conventional cages are also susceptible to coccidiosis, which contrasts with the traditional view that raising laying hens in conventional cages is unlikely to result in coccidiosis infection [10,15]. Hens raised in a cage system can still ingest enough oocysts from manure belts or edges of cages to induce clinical coccidiosis; however, the incidences are less [10,15]. Yet, there remains a dearth of knowledge regarding the effects of coccidiosis on laying hen performance, gastrointestinal health, oxidative stress, immune response, and skeletal health. However, broilers and laying hens have been intensively selected for many generations for different purposes (rapid growth and meat production vs. egg production), which might have changed their gastrointestinal physiology, immune response, and skeletal development. In this review, we aim to correlate findings from the broiler studies to best explain what might happen in laying hens infected with coccidiosis, particularly concerning gastrointestinal physiology, immune response, and potential mitigation strategies. 

## 2. Avian Coccidiosis

Avian coccidiosis is caused by multiple species of the genus *Eimeria* belonging to the subkingdom protozoa and phylum Apicomplexa [5]. *Eimeria* is an intracellular parasite that infiltrates intestinal enterocytes at different locations, depending on the species. Only seven have been recognized to infect chickens among the hundreds of *Eimeria* species including the following: *E. acervulina*, *E. brunetti*, *E. maxima*, *E. necatrix*, *E. mitis*, *E. praecox*, and *E. tenella* [2,5]. Among these *Eimeria* species known to infect chickens, *E. brunetti*, *E. maxima*, *E. necatrix*, *and E. tenella* are considered highly pathogenic, whereas *E. acervulina*, *E. mitis*, and *E. praecox* exhibit low pathogenicity (Table 1) [2,5]. In the past, there were sporadic reports of outbreaks of *E. hagani* and *E. mivati* in chickens. However, their existence was doubted, and they are now recognized as nomina dubia [16].

Avian coccidiosis is a common and widespread disease of chickens around the world and is transmitted by the fecal–oral route. Outbreaks of coccidiosis are reported wherever chickens are raised [2,5]. *Eimeria* oocysts can remain viable for a long period of time, and the disease can become endemic to the areas where environmental and managemental conditions favor year-round survival and multiplication [2,5]. The incidences of coccidiosis are usually higher in litter-based intensive systems, which favor the survival and accumulation of oocysts [4]. 

*Eimeria*’s life cycle is short and has high reproductive potential with self-limiting infection [2,5]. The life cycle of the *Eimeria* consists of an exogenous phase involving the sporulation of the oocyst in the environment and an endogenous phase involving asexual reproduction (schizogony) followed by sexual reproduction (gametogony) within the avian intestine. Infection initiates with the ingestion of sporulated oocysts from the environment [2,5,17]. Each sporulated oocyst contains four sporocysts, each containing two sporozoites. Upon ingestion, mechanical and biochemical forces break down the oocyst, releasing the sporozoites in the digestive tract [2,5]. Subsequently, sporozoites migrate to specific sites depending on the species involved and invade the intestine’s epithelial enterocytes. Within enterocytes, sporozoites transform into trophozoites and begin multiple asexual multiplications, forming schizonts, known as schizogony. Mature schizonts release merozoites, which again invade epithelial enterocytes, repeat the asexual cycle, and complete the second schizogonous cycle. Depending on the species, they might complete another schizogonous cycle before forming male (microgametocytes) and female (macrogametocytes) gametocytes [2,5]. Mature gametocytes release microgametes that fertilize mature macrogametocytes, resulting in the formation of oocysts. Upon maturation, oocysts rupture epithelial cells, which are excreted in feces and, under favorable conditions, sporulate within 48–72 h, becoming infective [2,5]. With each successive cycle, the number of sporulated oocysts increases exponentially in the environment, which retains their infectivity for extended periods [18]. The development of *Eimeria* in a host results in the destruction of the host’s intestinal tissues, leading to clinical manifestation observed in a disease outbreak. 

## 3. Host–Pathogen Interaction and Immune Responses against Coccidiosis

*Eimeria* species are highly host-specific and, once infected, can produce protective immunity against that particular species [19,20,21]. Notably, no cross-immunity is observed among *Eimeria* species. Generally, a large number of *Eimeria* oocysts are required to induce complete protective immunity, except for *E. maxima*, which is considered more immunogenic than other species [22]. The asexual life cycle of *Eimeria* is more immunogenic than the sexual life cycle. However, for the development of complete immunity and protection, both sexual and asexual phases of the life cycle are equally essential, especially for adaptive immunity [23,24].

Gut-associated lymphoid tissue (GALT) is an integral component of mucosal-associated lymphoid tissues and plays a vital role in the immune response as a first line of defense, preventing the progression of diseases and destroying infectious agents at an early stage [18,22]. GALT is a multilayered tissue with antigen-presenting cells, immunoregulatory cells, and effector cell types different from their systemic counterpart [19]. In poultry, during coccidiosis, GALT serves as antigen recognition and presentation, followed by the release of intestinal antibodies and activation of the cell-mediated immune response [18,19,25]. Various lymphoid tissues (Peyer’s patches, bursa of Fabricius, and cecal tonsils) have evolved in the GALT to produce verities of immune cells (epithelial, lymphoid, antigen-presenting, and natural killer cells) to protect against invading pathogens [18,19,22]. Peyer’s patches serve as antigen recognition and immune response activation, along with facilitating gastrointestinal IgA secretion [19,22,25]. Following this, activated B and T cells migrate to lamina propria, which serves as the effector site for immune responses, initiating antigen-specific/non-specific responses involving antibody production, leukocyte accumulation, and locally produced cytokines [19,25]. The activation of antigen-presenting cells, such as macrophages and dendritic cells, by coccidia upregulates the production of pro-inflammatory cytokines and chemokines from innate immune cells, which are essential for the development of the adaptive immune response. T lymphocytes play a crucial role in the response to coccidia infection, with various T cell subpopulations capable of recognizing multiple antigens and modulating humoral and cell-mediated immunity [18,19,25]. 

Cell-mediated immune responses are an important effector mechanism that includes antigen-specific and non-specific activation of various cell populations, including T lymphocytes, CD4+ helper T cells and CD8+ cytotoxic T cells, natural killer cells, and macrophages. T cell activation is associated with major histocompatibility complex (MHC), with cytotoxic T cells recognizing antigens presented in the context of MHC-I and helper T cells recognizing antigens associated with MHC-II molecules. Antigen-presenting and dendritic cells help activate naïve CD4+ T cells into different subsets such as Th1, Th2, Treg, and Th17 [19,26,27]. Activated helper T cells are involved in the humoral immune response, cytotoxic activity activation, macrophages, and natural killer cells [21]. Following coccidiosis, cytotoxic T cells, along with macrophages and natural killer cells, identify coccidia-infected host cells and modulate mononuclear cells to produce interferon-γ, activating proinflammatory pathways to inhibit the development of intracellular *Eimeria* within host cells [18,19,28]. Cytokines produced by CD4+ Th1 cells (IL-2, IL-12, IFN-γ, and TNF-α) aid in clearing the infection by promoting the activation of macrophages and other immune cells capable of eliminating intracellular coccidia [26,27]. The importance of cytotoxic T cells during coccidiosis has been demonstrated previously, as evidenced by the presence of intestinal intraepithelial lymphocytes expressing more than 75% CD8+ T cells, the presence of CD8+ cells in GALT within 24 h of infection, and the presence and activation of mono and polynuclear cells [19,22,29,30].

## 4. Coccidiosis and Its Effects on Pullets and Laying Hens

### 4.1. Gut Health and Oxidative Stress

The negative effect of coccidiosis on the parameters associated with gastrointestinal health, such as increased intestinal lesion scores, gastrointestinal permeability, a breach in tight junction integrity, damage to intestinal morphology, inflammation, and oxidative stress, are related to *Eimeria* multiplication and release in the gut lumen [2,5,6,7,31]. Continuous intestinal epithelial cell turnover occurs throughout the lifetime of the chickens. However, coccidiosis infection increases epithelial cell turnover by two times compared with noninfected birds [32]. It has been reported that the highest turnover of epithelial cells was observed between 5 and 7 days post-*Eimeria* inoculation due to the release of large numbers of first- and second-generation merozoites into the lumen. As a result, the mRNA and protein expression of a cell proliferative marker, the proliferating cell nuclear antigen, in the duodenum epithelium was upregulated, thus increasing crypt depth and resulting in a lower villus height to crypt depth ratio [33]. The difference between *Eimeria*-infected and non-infected birds in terms of villus height might range from 10 to 40% and is also dependent on the *Eimeria* species and level of infection [7,34,35,36,37,38,39,40]. This epithelial cell turnover during *Eimeria* infection might be a host defense mechanism to limit the multiplication of the *Eimeria* by the expulsion of infected cells [41]. Moreover, the tight junction proteins maintaining the tight junction integrity of the small intestine are composed of claudin, occludin, junctional adhesion molecules, and zonula occludens families [42]. These tight junction proteins were reported to be upregulated in *Eimeria*-infected birds along with increased permeability by more than 100%, supporting the observation of a rapid turnover rate in the intestinal epithelium during *Eimeria* infection [6,7,37,38,43]. Similar results were observed in pullets and laying hens of different age groups and at various stages of egg production [34,35,36].

During coccidiosis, the equilibrium between reactive oxygen species (ROS) and the host’s ability to neutralize ROS is disrupted, leading to oxidative stress. These ROS have a high affinity for the phospholipid bilayer of the cell, initiating lipid peroxidation and cytotoxic changes, which in turn damages the intestine, altering the gastrointestinal integrity and causing abnormal changes in the intestinal morphology [44,45]. Under normal circumstances, the production of ROS and its effects on cellular mechanisms are controlled by the host defense mechanism consisting of enzymes such as glutathione (GSH), glutathione peroxidases (GPx), and catalase [44]. During *Eimeria* infection, although oxidative defense mechanisms are active, the production of ROS exceeds the production of enzymes suppressing ROS, aggravating the situation. Previous studies have reported that *Eimeria* infection increased the markers associated with oxidative stress, such as reduced concentration of total antioxidant capacity (TAC) from 15 to 40% [31,46] and reduced GPx and GSH activity [31,47]. Furthermore, *Eimeria* infection also increased the markers of radical-induced damage, such as superoxide dismutase (SOD) and malondialdehyde (MDA) [34,35,36,44,48,49].

### 4.2. Growth Performance

The effect of coccidiosis on the growth performance of birds depends on the *Eimeria* species and the severity of the infection. Previous studies have reported that broilers challenged with 10^5^ oocysts of *Eimeria acervulina* reduced their body weight gain (BWG) by 10.3%, whereas 10^6^ oocysts per bird decreased BWG by 26.7% [50]. Likewise, similar results were observed with *E. maxima* and *E. tenella* [50]. On average, coccidiosis reduced the body weight (BW) of the chickens by 10%, irrespective of the species involved in the infection [51]. *E. maxima*-induced coccidiosis was observed to have the most severe negative impact on the BW of broilers, ranging from 23% to 37% [6,51], followed by *E. tenella*, ranging from 15% to 27% [52,53], and *E. acervulina*, from 16% to 19% [54,55]. However, Choi et al. (2021) [56] did not observe any reduction in the BW of broilers when they were inoculated with sporulated oocysts of *E. tenella* ranging from 6250 to 50,000 [56]. In cases of mixed *Eimeria* (*E. maxima*, *E. tenella*, and *E*. *acervulina*) infections, the reduction in BW can range from 13% to 50% with an increase in *Eimeria* dosage from 6250 to 50,000 *E. maxima*, 6250 to 50,000 *E. tenella*, and 31,250 to 250,000 *E. acervulina* [7,37,46]. However, mixed *Eimeria* infection has been associated with higher mortality rates, reaching up to 47%, and reductions in BW ranging from 9% to 28% in laying hen pullets aged two weeks, 8% to 15% in pullets aged 15 weeks, and 9% to 11% in hens aged 25 weeks during the early phase of infection [34,35,36]. Most studies have reported reductions in BW during the early phase of infection (1–8 days post-*Eimeria* inoculation) but not during the recovery phase (8–14 days post-*Eimeria* inoculation). During the early phase of infection, the reduction in feed intake and damage to the intestinal linings are more pronounced compared with those in the recovery phase, where feed intake and intestinal damage recover [7,35]. Additionally, during the early phase, nutritional redistribution occurs towards the immune response to subside the infection [7,35,36]. This reduction in feed intake and nutritional redistribution are associated with reduced growth or body weight loss in pullets and laying hens. 

### 4.3. Production Performance

**Figure 1:**
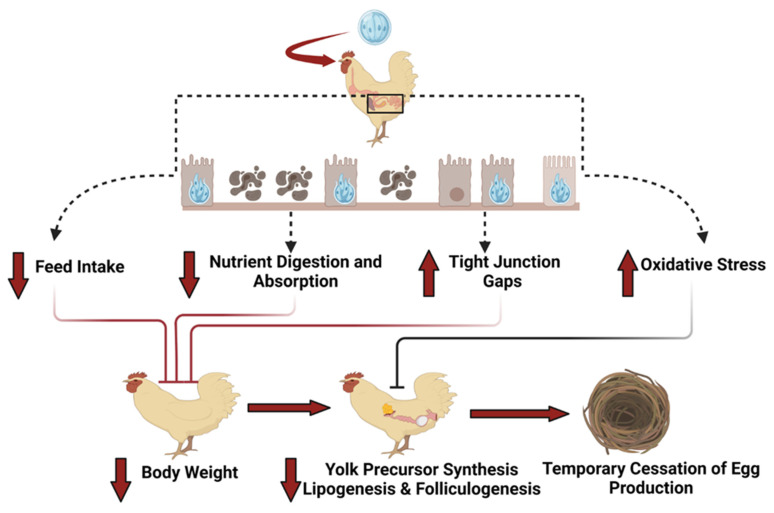
Potential mechanism of action showing how coccidiosis affects egg production in laying hens.

Coccidiosis rarely occurs in laying hens because of previous infection and the resulting immunity [10]. However, pullets reared in cages/on floors with coccidiostat drugs might not have enough exposure to coccidia to stimulate immunity. Outbreaks can occur after moving to layer facilities, either conventional cages or alternative housing systems [5,8,10]. Coccidiosis in laying hens has been reported during the early egg production period, around 23–24 weeks of age, and is characterized by high morbidity and mortality with a dramatic reduction in egg production [4,10,57,58,59]. Temporary cessation of egg production has been observed in laying hens infected with *E. maxima*, *E. acervulina*, and *E. tenella*. Hegde and Reid (1969) [57] reported that total egg production dropped to less than 20% when susceptible laying hens were independently challenged with different *Eimeria* species (*E. acervulina*, *E. maxima*, *E. brunetti*, *E. necatrix*, or *E. tenella*), with a significant drop observed two weeks post-challenge. It took 4–6 weeks to recover from infection, which varied among species. The mortality was more significant with *E. necatrix* and *E. maxima* following infection; however, the number of culled birds was not different from the control unchallenged group. Single-comb white leghorns infected with *E. mitis* experienced significant reductions in egg production and eggshell quality, undergoing complete molting before resuming egg production [58]. Hens recovered from coccidiosis may not reach their full egg-laying potential [60]. An outbreak of *E. necatrix* in a breeder hen facility resulted in significant mortality among breeder hens and roosters, as well as a 4.3% reduction in egg production following the recovery period compared with hens of the same age [59]. Recent studies in our lab have observed that coccidiosis at the pre-lay stage of growth delayed the onset of maturity and egg production by two weeks, and infection at peak egg production drastically dropped egg production and temporarily ceased egg production in some hens [34,36]. Reduced egg production during a coccidiosis outbreak might be because of the malabsorption of nutrients, including amino acids, carbohydrates, and minerals, which are essential during the laying period [6,7,8,61]. The possible mechanism of how coccidiosis affects the performance of laying hens is shown in Figure 1. Reduced feed intake and damage to intestinal linings following coccidiosis are evident. This damage caused by *Eimeria* to the intestinal tract affects the digestion and absorption of the nutrients required for egg formation [36]. Furthermore, it has been observed that plasma amino acids are lowered, and amino acid imbalance occurs during coccidiosis [55]. The limited availability of nutrients and amino acids in laying hens might reduce the synthesis of albumin, an important component of an egg, thus reducing egg production. Increased oxidative stress during coccidiosis might damage the developing oocytes and granulosa cells, leading to follicular atresia, production, and deposition of yolk precursors in selected ovaries, or occurrence of apoptosis in the oviducts and ovarian follicles [36,62,63]. These disruptions to normal physiological and reproductive functions might contribute to a decline in egg production. Additionally, the redistribution of energy during coccidiosis for tissue repair, inflammation, and maintaining oxidative status might also be responsible for the decline in egg production [36]. These studies showed that laying hens are susceptible to coccidiosis during the laying phase, with a dramatic reduction in egg production posing substantial challenges to the egg industry. The economic loss to the industry is mostly from reduced egg production, reduced feed intake, and an increased FCR due to malabsorption of nutrients and mortality.

## 5. Prevention and Control of Coccidiosis

### 5.1. Biosecurity and Management

Biosecurity is an important tool in poultry production to prevent disease outbreaks on farms. Once a coccidiosis outbreak occurs on a farm and the house becomes contaminated with oocysts, it is virtually impossible to completely decontaminate the environment [2,5]. Strict biosecurity measures should be implemented between farms and chicken houses to minimize the risk of cross-contamination and outbreaks. In litter-based intensive housing systems, *Eimeria* oocysts accumulate in large amounts and can remain viable for longer periods of time [2]. In those systems, management strategies should focus on maintaining the optimum level of moisture in the litter so that it can maintain the viable number of sporulated oocysts sufficient to generate protective immunity but not high enough to induce clinical disease. Furthermore, when employing vaccination as a preventive measure, it is also critical to maintain optimum litter moisture, as oocyst recycling is important in inducing a protective immune response against specific *Eimeria* species [64,65]. Additionally, immunosuppression from stressors such as heat, cold, or high stocking density in chicken houses might increase susceptibility to the disease; therefore, it is critical to maintain optimum environments in the houses [66].

### 5.2. Chemotherapy

Traditionally, chemotherapeutic (anticoccidial) drugs have been used in the poultry industry for years all around the world to control coccidiosis outbreaks in farms; however, the emergence of drug resistance among the zoonotic pathogens and concerns raised by the World Health Organization and consumers led to a ban of antibiotics use in food-producing animals including poultry as growth promoters and disease prevention [67,68]. In poultry, coccidiostat (inhibits the replication of *Eimeria*) or coccidiocidal (kills and destroys the *Eimeria*) drugs are used, which can be either synthetic or ionophores [64,68]. Synthetic anticoccidial drugs (chemicals) are produced by chemical synthesis from other chemical compounds that have diverse modes of action in controlling *Eimeria*, from inhibiting mitochondrial respiration and disrupting energy production (clopidol or decoquinate) to competitive inhibition of thiamine uptake (amprolium) or inhibiting folic acid pathways (sulphonamides). Some of the synthesized chemicals approved to be used in the poultry industry are diclazuril, halofuginone, clopidol, robenidine, decoquinate, nequinate, amprolium, and zoalene [68,69]. These chemicals act on the intracellular stage of *Eimeria* after they invade the host intestine and interfere with one or more stages of the life cycle [69]. However, while using these chemicals, it is important to consider that they are more susceptible to the development of resistance [68]. Additionally, these chemicals vary in their mode of action, efficacy, dose range, and susceptibility to resistance; it is important to rotate between them at certain intervals to minimize the development of resistance [68]. On the other hand, ionophores are fermented products from bacteria such as *Streptomyces* spp. and *Actinomadura* spp. [64,68]. Ionophores facilitate the transportation of ions across cell membranes, increasing the concentration of intracellular ions and disrupting the normal ion balance across cell membranes [70]. The common ionophores used in the poultry industry are monensin, narasin, salinomycin, maduramicin, semduramicin, and lasalocid. Even though these ionophores are not of human importance, the ban on antibiotics in animal production may include these, which have been the backbone of coccidiosis prevention programs for many years, forcing the poultry industry to look for alternatives [68]. Although the poultry industry has used shuttle or rotational programs to minimize resistance to these drugs in the past, most field strains of *Eimeria* have shown varying levels of resistance to more than one drug [64,71]. 

### 5.3. Vaccination

Vaccination against *Eimeria* species has become one of the most widely used methods in controlling coccidiosis outbreaks in the United States. The discovery of the self-limiting infection of *Eimeria* species and the development of resistance to reinfection by the same *Eimeria* species by Beach and Corl. (1925) [72] paved the way for the development and use of a live vaccine to control coccidiosis in the USA by 1952. In addition to self-limiting infection, *Eimeria* species are less susceptible to younger chicks, and the generation of acquired immunity against *Eimeria* species with limited to no pathogenesis made vaccination an efficient strategy to control coccidiosis in commercial poultry production [73]. The objective of immunization is to expose the birds to *Eimeria* species to elicit protective immunity against them, which is adequate to prevent future infections [9,15,65,66,74]. However, the major disadvantage of vaccination is the absence of cross-immunity among different *Eimeria* species; thus, it is more labor-intensive to prepare and has a high cost because of including multiple species in the vaccine [75]. When vaccinating meat birds against coccidiosis, the emphasis is placed on *E. acervulina*, *E. maxima*, and *E. tenella*. In contrast, for egg-laying hens, the focus shifts to *E. tenella*, *E. acervulina*, *E. maxima*, *E. brunetti*, *E. necatrix*, and *E. praecox*, primarily because of variations in the life spans of the respective hosts (Table 2) [15]. To ensure birds build strong immunity against *Eimeria* species, they must undergo oocyst recycling through three to four successive re-infections so that they have complete immunity against *Eimeria* by 3–4 weeks of age when most coccidiosis outbreaks occur [17,65,75,76]. In the United States, two types of live vaccines are available based on the pathogenicity of the parasites, including (i) nonattenuated and (ii) attenuated live oocyst vaccines [65,75]. Nonattenuated vaccines contain laboratory or field strains of *Eimeria* oocysts that have their virulence preserved to elicit protective immunity. With a nonattenuated live oocyst vaccine, immunity against *Eimeria* is produced by completing the life cycle and boosted by recycling oocysts from the litter for reinfections [9,17,65,73,75,76,77]. However, the significant disadvantages of nonattenuated vaccines are their short shelf life, high cost of production due to the inclusion of all *Eimeria* species, occasional outbreaks of coccidiosis on farms, halted growth, and a possible lack of compensation for growth after recovery [9,15,65,75,77]. On the other hand, live attenuated oocyst vaccines are selected via passage through embryonated hen eggs or by selection for precociousness and have demonstrated reduced pathogenicity while maintaining the ability to induce protective immunity [15,65,75,78]. This selection for precocious has decreased pathogenicity while still having the capacity to stimulate the immune response against selective *Eimeria* species [65,73,74,75,77]. Recombinant or subunit vaccines are composed of immunogenic antigens capable of producing protective immunity against *Eimeria*. However, identifying the antigens that elicit protective immunity and limited to no cross-immunity among *Eimeria* species are limiting factors for the development of recombinant vaccines [65,74,77]. Thus, for complete protection against the prevalent *Eimeria* species, it is essential to identify antigens against several isolates of the same species and for multiple species to ensure complete protection [65,77].

## 6. Nutritional Intervention for Coccidiosis

### 6.1. Role of Vitamins

#### 6.1.1. Vitamin D 

Vitamin D is a fat-soluble vitamin, and the active metabolites of vitamin D play an intrinsic role in calcium and phosphorus homeostasis and bone mineralization [79,80,81,82]. More recently, the immunomodulatory roles of vitamin D in improving the host defense against invading pathogens have been described [83,84,85,86]. The absorption of vitamin D is fat-dependent and undergoes hydroxylation into 25-hydroxyvitamin D3 [25(OH)D_3_] in the liver [86,87]. Subsequently, 25(OH)D_3_ is converted into its active form, 1,25-dihydroxy vitamin D [1,25(OH)_2_D_3_], in the kidney by the enzyme 25-hydroxyvitamin D-1α-hydroxylase. The hydroxylation of 25(OH)D_3_ to 1,25(OH)_2_D_3_ is not limited only to the kidney but also occurs in the bone, breast, and thigh muscles, small intestine, and immune cells [88]. 

The inclusion of 25(OH)D_3_ at varying levels in either layer pullets or turkey poults challenged with *Eimeria* reduced BW loss and fecal oocyst shedding, increased macrophage nitric oxide (NO) production, stimulated the activity of innate immune cells, and modulated adaptive immunity (CD4+, CD8+ and CD4+CD25+ T cells) and inflammatory cytokines (IL-1β and IL-10), as well as maintained tight junction [85,86,89]. Furthermore, supplementation of either vitamin D or 25(OH)D_3_ or in combination has been shown to improve the FCR and BWG in broilers vaccinated against *Eimeria* spp. at high doses; however, no beneficial effects were observed in reducing intestinal lesions and improving morphometry [90,91,92]. In broilers challenged with either *E. maxima* or mixed *Eimeria* spp. and fed reduced calcium/phosphorus diets, supplementation of 25(OH)D_3_ at the rate of either 3000 IU/kg or 4000 IU/kg has been shown to improve bone mineralization [83,84,93]. Furthermore, in laying hens challenged with lipopolysaccharides, 25(OH)D_3_ supplementation improved egg production by improving follicular development and oocyte maturation by increasing the expression of VDR in oocytes, maintaining the plasma estradiol/progesterone and luteinizing hormone levels [94,95,96,97]. Moreover, the lipopolysaccharide challenge increased the immune response (CD4+CD25+ T cells, proinflammatory cytokines, and plasma IgM levels) and oxidative stress (SOD, TAC, and GPx) in laying hens, which were normalized by the addition of 25(OH)D_3_ [97,98]. Previous studies have reported that inflammation and oxidative stress associated with coccidiosis are responsible for at least a 10% reduction in bone volume, bone mineral content and density, and osteoclastic activity [46,48,99]. These results suggest that vitamin D and its metabolites can potentially reduce the negative impacts of coccidiosis related to growth performance, egg production, oxidative stress, and bone mineralization.

#### 6.1.2. Vitamin E

Similar to vitamin D, vitamin E is a fat-soluble vitamin with a potent antioxidant capacity to protect cells and tissues from lipoperoxidation damage induced by reactive oxygen species [100,101,102]. Vitamin E from diets gets absorbed and incorporated into cell membranes to protect unsaturated fatty acids inside and outside the cells from reactive oxygen species. Furthermore, vitamin E prevents the excessive generation of reactive oxygen species from respiratory processes [103,104]. In addition, vitamin E is also involved in reducing inflammation, production of cytokine (TNF-α and IL-8) protecting cells of immune responses (lymphocytes, macrophages, and plasma cells), and enhancing the proliferation and functions of immune cells [100,105]. 

A previous study by Colnago et al. (1984) [106] observed that supplementation of 100 IU/kg vitamin E (DL-α-tocopheryl acetate) in a broiler diet and subsequent challenge by *E. tenella* or *E. maxima* reduced mortality, improved weight gain and the FCR, and reduced lesion scores. Similar results were observed when broiler chicks were challenged twice at 10 and 38 days of age and supplemented with both vitamin A (8 g/kg) and E (300 mg/kg) [107]. However, Allen and Fetterer (2002) [101] conducted two consecutive studies with increasing levels of vitamin E (13–200 IU/kg) in broilers challenged with *E. maxima* and did not observe significant differences in performance except for a slight reduction in lesion score. In broilers fed vitamin E at either 40 or 80 IU/kg alone or in combination with arginine and challenged with mixed *Eimeria* spp., heterophil and monocyte oxidative bursts (ROS production) and NO production decreased at seven days post-inoculation. They also observed lower lesions in challenged birds fed vitamin E, and birds fed a high level of vitamin E (80 IU/kg) in combination with arginine had higher levels of humoral antibodies IgG, IgM, and IGA [108]. Increased humoral and cell-mediated immune response with lower inflammatory mediators were observed in laying hens challenged with *Salmonella* enteritidis and fed diets supplemented with 30 IU/kg vitamin E [109]. Furthermore, da Silva et al. (2011) [110] observed that the inclusion of vitamin E at the rate of 65 mg/kg of diet increased the cell-mediated immune response of chickens vaccinated against coccidiosis and New Castle disease as measured by the cutaneous basophil hypersensitivity test. The antioxidant status (TAC, MDA, GPx activity, or SOD) and plasma lipid peroxidation of the broilers were improved following the *Eimeria* challenge in broilers, but the effects were not enough to improve performance compared with non-challenged broilers [111,112]. Furthermore, vitamin E has been shown to enhance the phagocytic activity of macrophages in chickens against pathogens [100]. 

In the case of egg layers, vitamin E inclusion has increased egg production by enhancing follicular development by increasing the concentration of reproductive hormones such as follicle-stimulating hormone, luteinizing hormone, estrogens, and progesterone [102]. These results from previous studies showed that vitamin E can be used in laying hens to improve their performance and boost the immune response; however, further studies are needed to confirm the effective dose.

#### 6.1.3. Other Vitamins

Vitamin A is a fat-soluble nutrient constituting a broad range of retinoid compounds and plays a crucial role in various physiological processes, including vision, growth, cell growth and differentiation, reproduction, immunity, skeletal development, and antioxidant properties [113,114,115]. The association between vitamin A deficiency and the increased incidence and severity of coccidiosis was noted as early as 1945 [116]. Dietary supplementation of vitamin A above the 1960 NRC recommendation (8000 IU/lb) has been shown to enhance broiler performance and the recovery of chicks infected with *E. tenella*, *acervulina*, or *necartix* and results in an improved performance after recovery [117,118]. Furthermore, Dalloul et al. (2002) [113] reported that vitamin A deficiency compromised the intestinal defense mechanism against *E. acervulina* infection, as evidenced by a reduced population of intraepithelial lymphocytes (CD4+ and CD8+ T cells) through alterations in concanavalin A-induced spleen lymphocyte proliferation. Moreover, vitamin A supplementation (12,000 IU/kg) enhanced intestinal morphometry and tight junction integrity in broiler chickens co-infected with *Clostridium* and *Eimeria* [119].

Unlike vitamins A, D, and E, vitamin C is a water-soluble vitamin with a strong antioxidant capacity and acts as a cofactor for collagen biosynthesis, thus maintaining epithelial barrier function and stimulating wound healing [120]. Moreover, it enhances innate immunity by increasing the phagocytic activity of mononuclear cells and adaptive immune responses by differentiation and proliferation of B- and T-cells [121]. Supplementing vitamin C (110–220 ppm) improved weight gain, increased feed intake, reduced mortality, and lowered the corticosterone level and heterophil: lymphocyte ratio in the blood [122,123]. Additionally, concurrent supplementation of vitamin C and protease improved mucin and NO production [124], and vitamin C and arginine or vitamin C and vitamin E in different experiments improved oxidative status but were not able to improve performance in *Eimeria*-infected broilers [112]. The effect of supplementing different vitamins on minimizing the effect of coccidiosis is summarized in Table 3. A hypothesis for using vitamins during coccidiosis could posit that both fat-soluble and water-soluble vitamins may be readily absorbed by the body, potentially exerting beneficial effects on modulating immune responses despite reduced feed intake and nutrient utilization. While there is a notable gap in the available literature concerning the utilization of vitamins to support laying hens during disease conditions, the positive outcomes observed in broilers suggest that similar benefits might extend to laying hens as well. However, further studies are necessary to confirm the optimal dosage for this beneficial effect in laying hens.

### 6.2. Role of Functional Amino Acids

#### 6.2.1. Arginine

**Figure 2:**
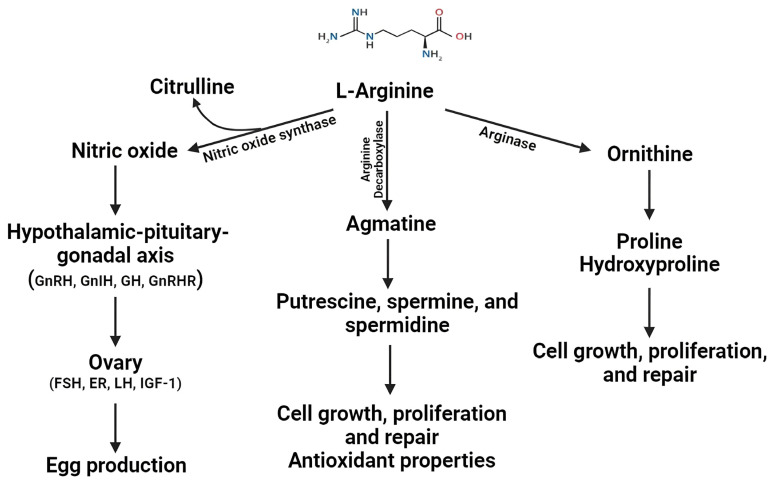
Effect of L-arginine and its metabolites on gut health and reproductive functions in laying hens. FSH: follicular-stimulating hormone; ER: estrogen; LH: luteinizing hormone; IGF: insulin-like growth factor 1.

Arginine, an essential functional amino acid, is vital in protein synthesis and accretion in chickens. Additionally, it contributes to secondary functions such as immune modulation, wound healing, and antioxidant activity [126,127,128,129]. In response to inflammation, arginine is metabolized to nitric oxide (NO) by nitric oxide synthase, which serves as an immune modulator, antioxidant defense, and cytotoxic mediator for non-specific host defenses [126,127,128]. Additionally, metabolites derived from arginine, such as proline, hydroxyproline, and polyamines, have been demonstrated to promote cell proliferation (Figure 2). Furthermore, ornithine and proline are primary amino acids found within collagen that are essential for wound healing and skeletal growth [126,127,128,130,131]. 

The secondary functions of arginine during coccidiosis have been extensively investigated in recent years and have shown beneficial effects in chickens. An early study by Allen and Fetterer (2000) [132] reported that *E. acervulina* infection reduced the plasma concentration of arginine, whereas its metabolite NO was significantly increased. The hypothesis for using arginine above the requirement could be that additional arginine might aid in balancing amino acids in plasma and provide a surplus for secondary functions. However, supplementation of arginine (500 mg/kg) did not reverse the negative effect associated with *E. acervulina*, *maxima*, or *tenella* infection except for a reduction in oocyst shedding of *E. tenella* [133]. Increasing dietary arginine concentration above the NRC requirement improved gut health by increasing villus height and goblet cell count and increasing mTOR pathways for healing [134,135]. The intestinal integrity of infected birds was improved when supplementing arginine, ranging from 1.24 to 1.48%, as observed by the upregulation of tight junction protein, permeability, and intestinal morphology [136,137]. Furthermore, arginine supplementation also reduced the expression of TLR-4 and IL-1β in broilers along with modulating the humoral immune response (secretory IgG and IgA) in broilers challenged with 20× Coccivac-B vaccine [134]. In addition, Yazdanabadi et al. (2020a; b) [138,139] reported that arginine supplemented 25–50% over the requirement increased the improved BWG, NO, and proinflammatory concentration along with gut morphometry. Furthermore, supplementation of arginine above the requirements has been found to improve the oxidative status of chickens following *Eimeria* challenge by GPx activity, reducing MDA and maintaining the levels of GSH and TAC [136,137,140]. 

In the case of laying hens, there is a lack of evidence for arginine supplementation and *Eimeria* challenge; however, in lipopolysaccharide-induced immune suppression, arginine supplementation above the requirements reduced the expression of inflammatory cytokines such as IL-1β, IL-10, TLR4, and NF-κB [141]. Furthermore, NO, a metabolite of arginine, plays a significant role in the hypothalamus–ovarian axis and plays a vital role in laying hen performance as well [142,143,144]. Based on previous studies in broilers and laying hens, supplementation of L-arginine above the requirements can be used in laying hens challenged with *Eimeria* to alleviate the symptoms associated with it.

#### 6.2.2. Methionine

Methionine is considered the first limiting amino acid in corn- and soy-based diets for laying hens and broilers and the first amino acid to be incorporated into the polypeptide chain during translation [126,145]. Aside from protein synthesis, methionine has antioxidant functions, acting as a precursor of cysteine, which is essential for GSH synthesis [128,146]. Both GSH and cysteine protect the cells against ROS [146]. Because of its role in balancing oxidative status through GSH and cysteine, its efficacy has been tested in broilers exposed to coccidiosis. A study by Pourali et al. (2014) [147] reported that supplementing 150% of total sulfur amino acids improved BWG, oocyst shedding, and hepatic malondialdehyde concentration; however, no effect was observed in GPx in broilers challenged with mixed *Eimeria* species. A study by Lai et al. (2018, 2023) [148,149] reported that supplementing extra methionine above the requirement in vaccinated birds did not have any beneficial effect on the performance, immune response, or oxidative status of Partridge shank broilers. However, reducing the level of methionine in the diets during *Eimeria* infection significantly affects the performance, oxidative status, and immune responses in challenged broilers [31,47,150,151], and supplementing 0.75% methionine in low-crude protein diets increased the detrimental effect of *Eimeria* infection on broiler health and performance [6]. In laying hens under heat stress, similar results were observed as those for *Eimeria*-challenged broilers, where reducing the methionine level reduced the production performance and bone mineralization and increased the oxidative stress of laying hens [152]. The role of TSAA is evident in maintaining performance, immune response, and oxidative status during *Eimeria* infection; however, it is still unclear if supplementing methionine above the requirement has a beneficial effect on laying hens under coccidiosis.

#### 6.2.3. Other Amino Acids

Glutamine, a conditionally essential amino acid, is the primary fuel for immune and intestinal epithelial cells and a precursor for GSH synthesis [153]. Supplementation of glutamine (0.5% or 1%) in coccidia-challenged broilers has been shown to reduce the expression of inflammatory cytokines IL-10 and IFN-γ, whereas it improves intestinal integrity and morphometry [154]. Furthermore, supplementing 0.75% glutamine in low-crude protein diets improved BWG during the recovery phase and maintained the expression of Claudin-1 compared to that of a normal protein diet in *Eimeria*-infected broilers [6]. 

Threonine is another essential amino acid and is essential for mucin and antibody (IgA, IgM) production in the gastrointestinal tract [155]. The intestinal mucus layer acts as the first line of defense against invading pathogens [155]. Therefore, factors increasing mucin production or intestinal secretions, as observed in coccidiosis, might increase threonine requirements during infection [156]. Supplementing 124% of threonine to its requirement in broilers challenged with mixed *Eimeria* species improved performance to the level of non-challenged birds, intestinal morphometry, oocyst shedding, and humoral immune response as measured by higher antibody production [157]. In contrast, threonine deficiency worsened the effects of coccidiosis in broilers challenged with coccidiosis by impairing intestinal morphometry and integrity, inflammatory responses, and lymphocyte population [158]. The effect of supplementing functional amino acids on the performance and intestinal health of chickens under coccidiosis infection is summarized in Table 4. Although these functional amino acids have shown beneficial effects in broilers under coccidiosis and in laying hens under normal conditions, their beneficial effect in laying hens under diseased conditions has not been fully explored. These amino acids have shown potential in mitigating the negative effect of coccidiosis in broilers and might have the same functions in laying hens, which need to be explored.

## 7. Role of Phytogenic Feed Additives

Phytogenic feed additives (PFAs) are plant-derived natural bioactive compounds or products that, when fed to animals, have a beneficial effect on performance and health [161]. Phytogenic feed additives have a wide range of bioactive compounds with antimicrobial, antioxidant, or anti-inflammatory properties and are used in traditional human medicines [161,162]. Phytochemicals are generally recognized as safe in the United States, indicating their safety for consumption. This designation strengthens the potential utilization of phytochemicals in poultry production for coccidiosis control. In the poultry industry, PFAs are gaining considerable attention mainly because of improvements in the performance of birds by improving gastrointestinal health alongside antioxidative and immunomodulatory effects [161]. The efficacy of various phytogenic feed additives, including *Artemisia annua*, curcumin, oregano, thyme, and their essential oils, has been investigated in broiler chickens infected with coccidiosis, demonstrating some beneficial effects. 

Dietary inclusion of curcumin powder (100–200 mg/kg) has been shown to reduce the lesion score and improve the oxidative status of broilers challenged with mixed *Eimeria* spp. [43]. Furthermore, essential oils of oregano have been shown to improve BWG and the FCR, reduce intestinal lesions and oxidative stress, and improve the gut morphology in broilers infected with coccidiosis [163,164]. The beneficial effects of these PFAs include supporting the host by immunomodulatory effects and providing protection against free radicals by scavenging reactive oxygen species and interfering directly with parasitic metabolism, reducing oocyst shedding and cecal short-chain fatty acid production [45,163,165]. Furthermore, Felici et al. (2023) [166] reported that bioactive compounds from a PFA can inhibit the intracellular replication of *Eimeria* and reduce schizont numbers. 

Artemisinin, a bioactive flavonoid found in *Artemisia annua* leaves (AA), has been shown to inhibit the growth of several stages of *Plasmodium* spp. [167,168]. The use of AA and its extract artemisinin has been shown to demonstrate anticoccidial effects against *E. tenella* [169,170,171,172,173] when infected alone. The use of either dried AA leaves or artemisinin has been shown to improve lesion scores, reduce oocyst shedding and sporulation, and modulate the humoral and immune response in chickens [169,174,175,176,177,178]. The mechanism of action of artemisinin is promoting apoptosis of infected host cells, thus neutralizing parasites [172]. Furthermore, artemisinin from AA is able to alter the cell wall formation of the oocysts, leading to the death of developing oocysts and a reduced sporulation rate [170]. The use of phytogenic feed additives in laying hens infected with coccidiosis has not been studied. However, results from laying hen studies reported that PFA inclusion in diets improved the performance, immune response, and antioxidant status of laying hens [179,180,181,182,183]. Since coccidiosis mainly affects the performance of birds with immune suppression and increased oxidative stress, PFAs can be helpful in laying hens infected with coccidiosis, as in broilers.

## 8. Role of Prebiotics, Probiotics, and Symbiotics

### 8.1. Probiotics

Probiotics are selective nonpathogenic microorganisms that, when administered in adequate amounts, offer a beneficial advantage to the host and improve gut functions by altering the gut microflora and reducing pathogenic bacteria colonization in the gastrointestinal tract [184,185,186,187]. By selectively eliminating pathogenic microorganisms from the GI tract through competitive exclusion, probiotics promote the growth of beneficial bacteria. Additionally, their metabolites, including short-chain organic fatty acids and hydrogen peroxide, exhibit antimicrobial properties, effectively inhibiting the growth of pathogenic bacteria [188,189]. Probiotic microorganisms help develop immune components in the GI tract and innate or adaptive immune responses and modulate the phosphorylation of cytoskeletal and tight junction proteins, improving the intestinal barrier [188,190,191]. Some of the commonly used probiotics in the poultry industry include *Bacillus*, *Lactobacillus*, *Enterococcus*, *Bifidobacterium*, and *Lactococcus*, and yeasts such as *Aspergillus*, *Candida*, and *Saccharomyces* [188,192].

The inclusion of *Bacillus* spp. in diets has been shown to improve the performance, tight junction integrity of the intestine, and immune response as well as positively influenced the cecal microbiome of broilers challenged with coccidiosis [193,194,195,196]. Similar results were observed in birds fed lactobacillus-based probiotics, including reduced mortality and oocyst shedding, upregulation of intestinal integrity, increased intestinal intraepithelial lymphocytes expressing CD4+ and CD8+ cells, and antibody titers in broilers challenged with coccidiosis [197,198,199]. In the case of laying hens, the dietary inclusion of probiotics (*Saccharomyces*, *Pediococcus*, *Lactobacillus*, or *Bacillus*), either alone or in combination, has improved the performance, oxidative status, immune responses, intestinal morphometry, and microbial composition of ceca positively in a non-challenge model [197,200,201]. The effectiveness of probiotics (*Bacillus amyloliquefaciens*, *Bacillus licheniformis*, and *Bacillus pumilus*) against *Salmonella* Enteritidis in laying hens and observed a significant reduction in salmonella colonization in ceca of mature laying hens [202,203].

### 8.2. Prebiotics

Prebiotics are non-digestible or selectively fermentable feed ingredients that, when incorporated into diets, are utilized by the host intestinal microbiota, selectively promoting the growth or activity of specific bacterial populations, positively influencing the microbiome, and improving the gut health of the host [18,204,205]. While selecting prebiotics, it is essential to consider (i) digestibility (non-digestible by host enzymes), (ii) absorption (should not be absorbed directly by host cells), (iii) selective fermentation by intestinal microbiota, (iv) selective promotion of the growth of beneficial bacterial population, and (v) stimulation of the immune response of the host [188,204,205,206]. Some of the prebiotics commonly used in the poultry industry are oligosaccharides (mannan-, galacto-, and xylo-oligosaccharides), β-glucan, and fructans [188,206,207,208]. 

The efficacy of prebiotics in improving the gut health of poultry has been investigated using both challenged and unchallenged models [209,210,211,212,213]. In experimentally infected broilers with coccidiosis, the administration of chitosan oligosaccharide (1 g/kg) improved various parameters, including BWG, FCR, oocyst shedding, intestinal tight junctions, and morphometry and reduced intestinal inflammation [214]. Similarly, broilers fed mannooligosaccharides (0.8 g/kg), xylooligosaccharides (0.5 g/kg), galactoglucomannan oligosaccharide (4%), or yeast cell wall polysaccharides (0.5 g/kg) exhibited improved performance, intestinal tight junction integrity and nutrient digestibility and mitigation of hostile cecal microbial populations and fermentation induced by *Eimeria* infection [209,210,211,215]. 

Feeding prebiotics such as dry whey powder (6 g/kg), mannan oligosaccharides (0.25–2 kg/kg), or fructooligosaccharides (0.25–1 g/kg) to laying hens has shown positive effects on their intestinal microbial populations. Specifically, it promotes beneficial bacteria like *Lactobacillus* spp. and *Olensella* spp. while reducing the abundance of harmful bacteria such as *Clostridium perfringens*, *Escherichia coli*, and *Salmonella enteritidis* [216,217,218]. Additionally, oligosaccharide supplementation has been linked to improved performance, digestibility, and upregulation of toll-like receptor-4, interferon-γ, and antibody production in laying hens [217,218].

### 8.3. Synbiotics

Synbiotics refer to the combined application of both prebiotics and probiotics, which exhibit synergistic effects on the host by enhancing gut health, the immune response, and microbial balance [219]. The rationale behind synbiotic uses lies in the premise that prebiotics facilitate the survival and colonization of probiotics in the host’s gastrointestinal tract, thereby positively influencing the health of the host [188,219]. In broiler chickens, both in ovo and in vivo studies have demonstrated the beneficial effects of synbiotics on performance, gut health, and the immune response, irrespective of whether the birds were challenged with coccidiosis or necrotic enteritis [220,221,222,223,224,225]. Similarly, in laying hens, supplementation with synbiotics containing both prebiotics and probiotics has been shown to enhance production performance, decrease levels of inflammatory cytokines, and increase populations of beneficial bacteria in the intestinal tract [226,227]. The efficacy of probiotics, prebiotics, and synbiotics in chickens infected with coccidiosis is summarized in Table 5. The effect of probiotics, prebiotics, and synbiotics in laying hens has been investigated in both normal and diseased (Salmonellosis) conditions and has shown positive impacts on the performance and wellness of hens. Although they have shown beneficial effects in broilers under coccidiosis, their effect in laying hens infected with *Eimeria* spp. hens has not been tested yet.

### 8.4. Postbiotics

In recent years, it has been established that the beneficial effects of probiotics are not only limited to when the microorganisms are alive but also after their death, leading to the development of new antibiotic alternatives, named postbiotics [234,235]. Postbiotics are non-viable bacterial cells or cell walls or metabolites derived from probiotics that have biological activity and offer physiological benefits to the host [235]. Different bioactive metabolites from probiotics with biological activity may include a range of compounds such as short-chain fatty acids, peptides, enzymes, polysaccharides, vitamins, cell surface proteins, and organic acids [235]. Recent studies conducted in chickens have shown that postbiotics confer beneficial effects on the host by positively modulating the intestinal microbiome, immune responses, and oxidative status [233,236,237,238]. In laying hen pullets challenged with *Salmonella* spp., dietary inclusion of postbiotics from *Saccharomyces cerevisiae* (1–1.5 kg/MT) or oral inoculation of *Lactobacillus* spp. postbiotics reduced the intestinal colonization of *Salmonella* [239,240]. Furthermore, *Lactobacillus*-derived postbiotics have been shown to enhance the performance, oxidative status, and intestinal morphology of broilers under heat stress [236,241]. Additionally, in broilers with experimentally induced necrotic enteritis, postbiotics from *Enterococcus* spp. *Pediococcus* spp., *Lactobacillus* spp., *Enterococcus* spp., and *Lactiplantibacillus* spp. were able to improve performance, lesion scores, and oxidative status and reduce proinflammatory responses [237,238,242]. Moreover, the inclusion of maltol (a metabolite of Bacillus subtilis) in a broiler diet infected with *E. maxima* was able to reduce intestinal damage and inflammatory response [233]. Although the use of postbiotics in broilers and laying hens infected with *Eimeria* spp. has not been extensively explored, previous studies have demonstrated their efficacy in reducing the symptoms associated with coccidiosis in other stress conditions. 

## 9. Conclusions

To conclude, this review summarizes the effect of coccidiosis on laying hen health and performance and its potential to cause a significant economic loss to the egg industry. Since the ban on antibiotics as growth promoters in animal production, there has been an increase in the incidences of economically important diseases in poultry, such as coccidiosis and necrotic enteritis. Several antibiotic alternatives (vitamins, functional amino acids, phytogenic feed additives, prebiotics, probiotics, synbiotics, and postbiotics) have been tested in broilers with positive effects on minimizing the effect of coccidiosis, but their beneficial effects have not been explored in laying hens. These nutritional strategies have been shown to have the potential to mitigate the negative effects of coccidiosis in laying hens as well. However, before applying these nutritional strategies either to boost immunity after vaccination or to mitigate the negative effects of coccidiosis, they need to be tested in laying hens. Additionally, while implementing these nutritional strategies to mitigate the adverse effects of coccidiosis, it is important to consider that these strategies are not capable of curing coccidiosis completely, and poultry producers must evaluate the severity of the conditions. Future studies should focus on evaluating nutritional strategies either alone or in combination, focusing on performance, gut health, and immune responses against coccidiosis in laying hens.

## Figures and Tables

**Table 1:** List of *Eimeria* species important in laying hens with their predilection sites, characteristic lesions, pathogenicity, and immunogenicity.

Species	Pathogenicity	Predilection Site in the Intestine	Characteristic Lesions	Immunogenicity
*E. mitis*	Low	Mid-intestine (Meckel’s diverticulum to cecal junction)	Mucoid exudate and no distinct lesions	Moderate
*E. praecox*	Low	Anterior intestine (duodenum)	Mucoid exudate and no distinct lesions	Moderate
*E. acervulina*	Low to moderate	Anterior intestine (duodenum)	Whitish round lesions	Moderate
*E. maxima*	Moderate to high	Mid-intestine (part of jejunum and ileum)	Petechiae hemorrhage and blood-tinged exudate	High
*E. brunetti*	Moderate to high	Posterior intestine (Meckel’s diverticulum to cecal junction)	Blood clot and coagulated necrosis	High
*E. necatrix*	High	Mid-intestine (part of jejunum and ileum)	Petechiae hemorrhage and blood-filled exudate	Low
*E. tenella*	High	Ceca	Hemorrhagic lumen and blood clot	Low

**Table 2:** Commercial vaccines available in the U.S. for use in laying hens; adapted from Price (2014) [15] and Cervantes (2023) [68].

Trade Name	*Eimeria* Species Present in Vaccine	Vaccine Type	Route of Administration	Age of Birds at Vaccination	Manufacturer
Coccivac-D2	*E. acervulina*, *E. brunetti*, *E. maxima*, *E. mivati*, *E. necatrix*, and *E. tenella*	Nonattenuated	Hatchery spray, ocular, drinking water, feed spray	Single dose at 1 to 4 d	Merck
Immucox C	*E. acervulina*, *E. brunetti*, *E. maxima*, *E. necatrix*, *E. tenella*, and *E. praecox*	Nonattenuated	Drinking water, oral gel	Single dose 1 to 4 d	Ceva
Paracox	*E. acervulina*, *E. brunetti*, *E. maxima*, *E. mitis*, *E. necatrix*, *E. praecox*, and *E. tenella*	Attenuated	Drinking water, feed spray	Single dose at 1 to 9 d	Merck
Immunocox5	*E. acervulina*, *E. brunetti*, *E. maxima*, *E. necatrix*, and *E. tenella*	Nonattenuated	Gel	Single dose 1 to 4 d	Ceva

**Table 3:** Effects of vitamin supplementation on performance and intestinal health of chickens infected with *Eimeria* species.

Nutritional Interventions	Breed	*Eimeria* Infection	Dosage	Impacts on Performance	Impact on Health	Reference
25-hydroxycholecalciferol	White Leghorn	*E. acervulina*, *E. maxima*, and *E. tenella* (Inovocox)	4000 IU/kg	Improved performance	● ↓ CD8+ cells. ● ↑ CD4+CD25+ cells. ● ↓ IL-1β expression. ● ↑ IL-10 expression.	[85]
Vitamin D_3_ + 25-hydroxycholecalciferol	Cobb 700	7× live vaccine (*E. acervulina*, *E. maxima*, and *E. tenella*)	2000–8000 IU/Kg	Improved BW, BWG, and FCR	● ↑ Duodenal morphology. ● ↑ Tibia breaking strength. ● ↑ Tibial bone mineralization.	[91]
Vitamin D_3_ or 25-hydroxycholecalciferol	Ross 308	*E. maxima* (7000)	4000 IU/Kg	Improved BW, BWG, and FCR	● ↑ Bone breaking strength. ● ↑ Bone mineralization. ● ↓ Jejunal morphology.	[84]
Vitamin D_3_ or 25-hydroxycholecalciferol	Ross 308	*E. maxima* (7000)	4000 IU/kg	No effect on performance	● ↑ Bone breaking strength. ● ↑ Ash content and percentage.	[83]
Vitamin D_3_ or 25-hydroxycholecalciferol	Cobb 500	2× live vaccine	1375 or 2750 IU/kg	Improvement in BW and FCR	● ↓ Intestinal morphology. ● ↑ Tibia breaking strength. ● ↑ Bone ash %.	[92]
25-hydroxycholecalciferol	Cobb 500	*E. acervulina* (62,500), *E. tenella* (12,500), and *E. maxima* (12,500)	3000 IU/kg	No effect on performance	● ↑ Bone ash. ● ↑ Bone mineral content of the birds. ● ↑ Tissue and bone volume of the femur.	[93]
DL-alpha tocopheryl acetate	Hubbard	*E. tenella* (150,000) or *E. maxima* (50,000)	100 IU/Kg	Improved BWG, FI, and FCR	● ↓ Mortality. ● ↓ Lesion scores. ● ↑ Packed cell volume.	[106]
DL-alpha tocopheryl acetate	Ross	*E. maxima* (175,000 or 40,000)	13–200 ppm	No effect on performance	● ↓ Lesion scores. ● ↑ Plasma NO.	[101]
DL-alpha tocopheryl acetate	Cobb 500	*E. acervulina* (100,000), *E. maxima* (60,000), and *E. tenella* (40,000): Coccivac-B	40 or 80 IU/kg	-	● ↑ Heterophil and monocyte oxidative burst. ● ↑ IgG, IgM, and IgA production. ● ↓ Lesion scores.	[108]
D-α-tocopherol	Ross 308	Livacox Q vaccination	65 mg/kg	-	● ↑ Cell-mediated immunity.	[110]
D-α-tocopherol	Ross 308	*E. tenella*	100, 316, or 562 mg/kg	-	● ↓ MDA concentration.	[111]
D-α-tocopherol and L-ascorbic acid	Cobb 500	100× Advent vaccine	80 mg/kg VE + 1 g/kg VC	Improved BWG	● ↑ Nitric oxide production. ● ↑ GPx activity. ● ↓ Lesion scores.	[112]
L-ascorbic acid	Vantress-White Plymouth Rock cross	*E. maxima* (30,000), *E. brunetti* (100,000), *E. necatrix* (30,000), or *E. tenella* (30,000)	110 mg/kg	No effect on performance	● Increased mortality.	[123]
L-ascorbic acid	Hubbard	*E. tenella* (300,000)	150 or 300 mg/kg	Improved BW, FI, and FCR	● ↓ Plasma corticosterone. ● ↓ Heterophil/lymphocyte ratio.	[122]
Vitamin A	Plymouth Rock		8000 IU/lb	Improved BWG	● ↑ Recovery.	[117]
Vitamin A	Ross 308	*E. acervlina* (10,000)	VE deficiency	-	● ↓ CD4+, CD8+ cells. ● ↑ Oocyst shedding. ● ↓ IFN-γ. ● ↓ Lymphocyte proliferation.	[113]
Vitamin A	Broilers	*E. maxima* + *Clostridium perfringens*	12,000 IU/kg	Improved BW, BWG, and FI	● ↑ Intestinal morphology. ● ↑ Expression of tight junction proteins.	[119]
Menadione sodium bisulfite	Lancaster X New Hampshire	*E. tenella* (500,000)	1.06 or 9.72 g/ton	-	● ↓ Mortality.	[125]

IU: international unit; BW: body weight; BWG: body weight gain; FI: feed intake; FCR: feed conversion ratio; NO: nitric oxide; IL-1β: Interleukin 1β; IL-10: Interleukin 10; IFN-γ: Interferon γ; IgA: immunoglobin A; IgG: immunoglobin G; IgM: immunoglobin M; ppm: parts per million; VE: vitamin E; VC: vitamin C; MDA: malondialdehyde; GPx: glutathione peroxidase.

**Table 4:** Effects of functional amino acids supplementation on the performance and intestinal health of chickens infected with *Eimeria* species.

AA	Breed	*Eimeria* Challenge	AA Dosage	Impact on Performance	Impact on Health	Reference
Arginine	SexSal	*E. maxima* (30,000), or *E. tenella* (50,000), or *E. acervulina* (500,000)	+500 or +1000 mg/kg	No effect on BWG	● ↓ Oocyst shedding of *E. tenella*.	[108]
	Cobb 500	*E. acervulina* (100,000), *E. maxima* (60,000), and *E. tenella* (40,000): Coccivac-B	Added 0.3% or 0.6% to make 1.74% and 2.04% (NRC, 1994)		● ↑ Heterophil and monocyte oxidative burst. ● ↑ IgG, IgM.	[108]
	Ross 708	20× Coccivac-B	106·4% and 160·8% of (NRC, 1994)	Improved BWG and FCR	● ↑ Villus height and crypt depth. ● ↑ Goblet cell counts and density. ● ↓ Expression of TLR4, IL-1β.	[141]
	Cobb 500	100× Advent coccidiosis vaccine	1.80% (NRC 1994)	No effect on BWG	● ↑ NO production. ● ↑ GPx activity. ● ↓ Intestinal lesions.	[135]
	Ross 308	*E. acervulina* (150,000), *E. tenella* (15,000), *E. maxima* (15,000), and *E. necatrix* (15,000)	105 and 110% (breeder recommendation)	No effect on BWG or FCR	● ↓ Crypt depth and oocyst shedding.	[135]
	Ross 308	*E. acervulina* (3.5 × 10^5^)	Low arginine (0.74%)	Reduced BWG, FI, and FCR	● ↓ Plasma Arg: Lys ratio.	[129]
			High arginine (1.23%)	Improved BWG, FI, and FCR	● ↑ Plasma Arg: Lys ratio. ● ↑ NO production.	
	Cobb 500	*E. acervulina* (62,500), *E. tenella* (12,500), and *E. maxima* (12,500)	100%, 108%, and 116% (breeder recommendation)	Improved BWG, FI, and FCR	● ↓ Gut permeability. ● ↑ Tight junction proteins. ● ↑ VH and reduced CD. ● ↑ NO production. ● ↓ SOD activity.	[136]
	Ross 308	*E. necatrix* (15,000), *E. maxima* (20,000), *E. acervulina* (15,000), and *E. tenella* (170,000)	125 and 150% (breeder recommendation)	Improved BWG, FI, and FCR	● ↓ IL-1β, IL-2, IL-6, IFN-γ, and TNF-α. ● ↑ NO production. ● ↓ Oocyst count.	[139]
	Cobb 500	*E. acervulina* (62,500), *E. tenella* (12,500), and *E. maxima* (12,500)	16% CP plus 0.75% ARG	No effect on performance	● ↓ Gut permeability. ● ↑ VH and AA digestibility.	[159]
	Cobb 500	*E. acervulina* (62,500), *E. tenella* (12,500), and *E. maxima* (12,500)	150% (breeder recommendatio)		● ↓ Gut permeability. ● ↑ Tight junction proteins. ● ↓CD8+:CD4+. ● ↑ NO production.	[140]
TSAA	Ross 308	750 *E. acervulina*, *E. tenella*, and *E. maxima*	150% TSAA (breeder recommendation)	Improved BWG	● ↓ Oocyst shedding. ● ↓ NO production. ● ↓ MDA concentration.	[147]
	Partridge Shank broilers	50,000 *E. tenella*	125% and 150% (breeder recommendation)	No effect on BWG; ADFI 150% methionine reduced BWG	● ↓ VH and lesion score. ● ↓ CD4+ and CD8+ cells. ● ↓ IFN-γ.	[151]
		100 × Advent vaccine (*E. acervulina*, *E. maxima*, and *E. tenella*)	0.6, 0.8, 0.9, and 1.0% TSAA	Below the requirement reduced BWG, ADFI, and gain: feed	● ↓ TSAA reduced IgA production.	[151]
	Cobb 500	*E. acervulina* (62,500), *E. tenella* (12,500), and *E. maxima* (12,500)	16% CP + 0.75% Met	Negatively affected BWG, FI, and FCR	● ↑ Oocyst shedding. ● ↓ Intestinal morphology.	[159]
Threonine	New Hampshire × Colombian	1500 *E. maxima*	125% of threonine (breeder recommendation)	Improved BWG, ADFI, and FCR	● ↓ IL-1β. ● ↑ IL-12.	[160]
	Ross 708	Coccivac^®^-B	61.25% (NRC, 1994)	Exaggerated performance	● ↑ Oocyst shedding. ● ↑ Gut permeability, IgA production. ● ↓ Intestinal morphology. ● ↓ Goblet cell counts. ● ↓ Lymphocytes expressing CD4+, CD8+, CD3 cells.	[158]
	Cobb 500	Mixed *Eimeria* oocysts	112%, 124%, and 136% (breeder recommendation)	Improved BWG and FCR	● ↑ Intestinal morphology. ● ↓ Oocyst shedding. ● ↑ IgG and IgM production.	[157]
	Cobb 500	*E. acervulina* (62,500), *E. tenella* (12,500), and *E. maxima* (12,500)	16% CP and 0.75% threonine	Improvement in BWG, FI, and FCR	● ↓ Oocyst shedding. ● ↑ Intestinal morphology. ● ↓ Claudin-1 expression.	[159]
Glutamine	Cobb 500	20 × dose of Coccivac B	0.5% and 1% glutamine	No effect on performance	● ↓ Inflammatory and proinflammatory cytokines (IL-10, IFN-γ). ● ↑ Expression of tight junction proteins. ● ↓ Intestinal CD and ↑VH.	[154]

AA: amino acid; TSAA: total sulfur amino acid; CP: crude protein; BWG: body weight gain; ADFI: average daily feed intake; FCR: feed conversion ratio; NO: nitric oxide; IL-1β: Interleukin 1 β; MDA: malondialdehyde; GPx: glutathione peroxidase; SOD: superoxide dismutase; TLR-4: toll-like receptor-4; IFN-γ: Interferon γ; IL-2: Interleukin 2; IL-6: Interleukin 6; IL-10: Interleukin 10; IL-12: Interleukin 12; VH: villus height; CD: crypt depth; IgG: immunoglobin G; IgM: immunoglobin M.

**Table 5:** Effects of prebiotics, probiotics, and synbiotics supplementation on performance and intestinal health of chickens infected with *Eimeria* species.

Nutritional Interventions	Breed	*Eimeria* Infection	Dosage	Impacts on Performance	Impact on Health	Reference
Galacto-glucomannan oligosaccharide-arabinoxylan	Ross × Ross	*E. acervulina* (1,000,000)	1, or 2, or 4%	Improved FI but not BW	● ↓ Propionate in ceca. ● ↑ IFN-γ, IL-1β, IL-6, IL-12 expression. ● ↑ *Bifidobacterium* spp., *Lactobacillus* spp. in ceca.	[209]
Mannan-oligosaccharides	Ross 308	*E. tenella* (20,000–30,000)	0.8 g/kg	Improved BWG, FI, and FCR	● ↓ Oocyst shedding. ● ↓ Cecal lesions.	[215]
Chitosan-oligosaccharide	Cobb 500	15× Coccivac^®^-B-52 (*E. acervulina*, *E. maxima*, *E. maxima MF*, *E. mivati*, and *E. tenella*)	1 g/kg	Improved BW, BWG, FI, and FCR	● ↓ Oocyst shedding. ● ↑ Intestinal morphology. ● ↑ Expression of tight junction protein. ● ↓ Expression of TNF-α, IFN-γ, and TLR-4. ● ↑ Expression of IL-6, IL-10, and IL-1β.	[214]
Nucleotide-rich yeast extract	Ross 708	*E. acervulina* (25,000) and *E. maxima* (5000)	0.5 g/kg	Improved BW, BWG, and FCR	● ↑ Jejunal morphology. ● ↓ Cecal SCFA concentration.	[210]
Xylo-oligosaccharides	Ross 308	12× Paracox 8	0.025%	Improved BWG and FI	● ↑ Propionic and butyric acid in ceca. ● ↓ %G+C profile of cecal bacteria.	[228]
Xylo-oligosaccharides	Cobb 500	*E. acervulina* (62,500), *E. tenella* (12,500), and *E. maxima* (12,500)	0.5 or 1 g/kg	No effect on performance	● ↓ Duodenal lesions. ● ↓ CLDN-1 overexpression. ● ↓ Branched-chain fatty acids.	[211,229]
Yeast cell wall	Ross 308	*E. tenella* (5000)	0.1 or 0.2%	Improved BWG, FI, and FCR	● ↑ Jejunal morphology. ● ↓ Bursa follicle length and area.	[230]
*Lactobacillus* spp.	Ross 308	*E. acervulina* (10,000)	1 g/kg	-	● ↓ Oocyst shedding. ● ↑ Intraepithelial lymphocytes expressing CD4, CD8 cells.	[199]
*Saccharomyces cerevisiae*	Broilers	*E. tenella, E. maxima*, and *E. necatrix* (70,000 oocysts total)	0.1, or 1, or 10 g/kg	Improved BWG and FCR	● ↑ Cellular immune response. ● ↑ IgM and IgG concentration. ● ↓ Oocyst shedding and lesion score.	[197]
*Lactobacillus salivarius* and *L. jhonsonii*			10^6^, or 10^7^, or 10^8^ CFU/L drinking water			
*Bacillus subtilis* 747	Ross 708	*E. maxima* (10,000)	1.5 × 10^5^ CFU/g feed	Improved BWG and FCR	● ↓ Oocyst shedding and lesion score. ● ↓ IL-1β, IL-6, IL-2, and IFN-γ. ● ↑ Expression of tight junction protein.	[195]
*Bacillus licheniformis*-A *Bacillus amyloliquefaciens*-B *Bacillus amyloliquefaciens*-D	Ross × Ross	*E. tenella* (5000), *E. maxima* (5000), and *E. acervulina* (5000)	1.5 × 10^5^ CFU/g of feed	Improved performance	● ↓ Oocyst shedding and lesion score; ● ↑ IL-6, IL-8, and IL-10. ● ↑ JAM-2.	[196]
*Bacillus subtilis* (BS-9)	Ross 708	*E. maxima* (25,000) and *E. acervulina* (100,000)	10^8^ CFU/bird/d in drinking water	No effect on BW, FI, and FCR	● ↑ Intestinal morphology. ● ↓ Bursa weight.	[231]
*Lactobacillus rhamnosus*	Yellow broilers	*E. acervulina, E. maxima*, and *E. tenella* (100,000)	10^6^, or 10^8^ CFU in ovo	-	● ↓ Internal organ growth. ● ↓ Intestinal morphology. ● Exaggerated coccidia infection.	[232]
*Lactobacillus plantalum* + *Shallot extract*	Broilers	*E. tenella* (20,000)	10^6^ cfu/mL of Lactobacillus plantalum + 2% Shallot extract in water	-	● ↓ Cecal lesion score.	[220]
*Lactobacillus reuteri, Enterococcus faecium, Bifidobacterium animalis, Pediococcus acidilactici*, and a fructooligosaccharide	Turkey poult	*E. adenoides* and *E. meleagrimitis*	-	Improved BWG	● ↓ Oocyst shedding and lesion score.	[221]
Postbiotics (Mantol)	Ross 708	*E. maxima* (10,000)	10 mg/kg	No effect on performance	● ↓ Oocyst shedding and lesion score. ● ↓ IL-1β, IL-6, IL-17, IL-10, and IFN-γ.	[233]

BW: body weight; BWG: body weight gain; FI: feed intake; FCR: feed conversion ratio; NO: nitric oxide; IL-1β: Interleukin 1-β; IL-6: Interleukin 6; IL-10: Interleukin 10; IL-12: Interleukin 12; TLR-4: toll-like receptor-4; IFN-γ: Interferon γ; TNF-α: tumor necrosis factor-α; SCFA: short chain fatty acid; CLDN-1: Claudin -1; IL-2: Interleukin 2; IL-8: Interleukin 8; JAM-2: junction adhesion molecule-2; CFU: colony-forming unit.
